# Reassessment of variants of uncertain significance in tumor suppressor genes using new ClinGen PP1/PP4 criteria guidance

**DOI:** 10.1038/s41431-025-01911-z

**Published:** 2025-07-23

**Authors:** Young-gon Kim, Changhee Ha, Ja-Hyun Jang, Mi-Ae Jang, Jong-Won Kim

**Affiliations:** 1 Department of Laboratory Medicine and Genetics, Samsung Medical Center, Sungkyunkwan University School of Medicine, Seoul, Republic of Korea; 2 Department of Laboratory Medicine, Konkuk University Medical Center, Konkuk University School of Medicine, Seoul, Republic of Korea

**Keywords:** Genetics research, Cancer genetics

## Abstract

Recently, new clinical genome resource (ClinGen) guidance focusing on cosegregation (PP1) and phenotype-specificity criteria (PP4) were introduced, based on the observation that the phenotype specificity could provide greater level of pathogenicity evidence. This study aimed to reassess variants of uncertain significance (VUS) found in tumor suppressor genes with specific phenotypes using these new recommendations. We retrieved VUS from an in-house database of all germline variants detected using sequencing since 2008. Patients carrying VUS from seven target tumor suppressor genes, *NF1*, *TSC1*, *TSC2*, *RB1*, *PTCH1*, *STK11*, and *FH*, were selected and the pathogenicity of each variant was reassessed using the new ClinGen PP1/PP4 criteria. In total, 128 unique VUS from 145 carriers were evaluated. Initial classification using the classic PP1/PP4 criteria from ACMG/AMP and point-based classification resulted in 21 variants being reclassified (2 pathogenic variants, 3 likely pathogenic variants [LPVs], 15 likely benign variants, and 1 benign variant), leaving 101 VUS. Applying the new ClinGen PP1/PP4 criteria, 32 (31.4%) remaining VUS were reclassified as LPVs. The reclassification rate was highest in *STK11* (88.9%). Representative cases highlighted successful reclassification owing to highly specific phenotypes aligned with the new criteria. The new ClinGen PP1/PP4 criteria significantly improved the reclassification of VUS in tumor suppressor genes associated with specific phenotypes. The new criteria could substantially enhance the accuracy of variant classification.

## Introduction

Due to the widespread adoption of next-generation sequencing (NGS), the number of sequence variants requiring correct interpretation has drastically increased, necessitating a systematic approach. Since its publication in 2015, the classification scheme of the American College of Medical Genetics and Genomics and the Association for Molecular Pathology (ACMG/AMP) has provided an internationally employed standard for assessing variants [1]. In addition, many disease- and criteria-specific recommendations have been published by the Clinical Genome Resource Sequence Variant Interpretation Working Group (ClinGen SVI) (https://www.clinicalgenome.org/working-groups/sequence-variant-interpretation/) to further refine the ACMG/AMP guidelines. Based on the observation that ACMG/AMP guidelines are compatible with a Bayesian classification framework [2], ClinGen SVI developed a quantitative scoring framework, abstracting ACMG/AMP evidence criteria into points [3]. This point-based adaptation of ACMG/AMP classification system has proven effective and has been adopted in recent publications [4–6].

One of the purposes of these refinements of the variant classification scheme was to reduce the number of variants of uncertain significance (VUS). The uncertainty associated with VUS adds complexity to clinical decision-making and can lead to harms and increased costs for patients and the healthcare system, including time-consuming interpretation, unnecessary treatments, and potential psychological distress [7–9]. Therefore, refining the variant classification aims not only to streamline interpretation but also to enhance clinical accuracy and reduce the risk of inappropriate management resulting from uncertain variant interpretation. Refinements to classification schemes, including those published by ClinGen SVI, can reduce the number of VUS by incorporating disease- or gene-specific knowledge into the interpretation [10**–**13].

In this regard, a new ClinGen guidance was published to help increase the applicability of the co-segregation criteria (PP1/BS4) and phenotype specificity criteria (PP4) of ACMG/AMP guidelines based on the important observation that the co-segregation criteria and phenotype specificity criteria are inextricably related to each other [14]. This new modification is based on the point-based classification scheme mentioned above, in which point ranges for each category are defined as ≥10 (pathogenic), 6–9 (likely pathogenic), 0–5 (VUS), −1 to −6 (likely benign), and ≤−6 (benign), where one, two, four, and eight points are given for each of supporting, moderate, strong, and very strong pathogenic evidences, respectively, and −1, −2, and −4 points are given for supporting, moderate, and strong benign evidences, respectively [3]. The main goal of this modification was to provide a systematic method to assign higher scores based on supporting evidence from the phenotype specificity criteria, when phenotypes are highly specific to the gene of interest. Extreme examples have been provided using genes with locus homogeneity in which only one gene could explain the phenotype [14]. In scenarios of locus homogeneity, up to five points can be assigned solely from the phenotype specificity criteria, based on the high contribution of the gene to the phenotype. Co-segregation criteria cannot be applied to genes with locus homogeneity because the co-segregation of any variant detected in the gene to the causative variant is predictable, given the high level of linkage disequilibrium caused by the short distance between the two variants [14]. Conversely, phenotypes with locus heterogeneity have multiple causative genes, and only lower scores are available for phenotype specificity criteria, based on lower contribution of each gene on the phenotype [14]. Extreme examples of locus heterogeneity may include intellectual disability and the use of PP4 criteria would be inappropriate without additional characteristic symptoms.

Many tumor suppressor genes are associated with characteristic phenotypes that minimally overlap with other clinical presentations, such as *NF1* and *FH*. When a VUS is found in one of these genes and the phenotypes are highly consistent, such as an *NF1* VUS detected in a patient with multiple café-au-lait spots and extensive neurofibromatosis, the VUS is highly suspected to be the actual cause of the disease, although rigorous application of the ACMG/AMP guidelines may fail to classify this variant as likely pathogenic. Given the emerging targeted therapies for patients with *NF1* pathogenic variants (PVs) and the widespread availability of preimplantation genetic diagnosis, the proposed classification system that assigns higher scores to phenotype specificity criteria would benefit many potential patients [15, 16].

This study aimed to reassess previously reported VUS from seven tumor suppressor genes associated with specific phenotypes, *NF1*, *TSC1*, *TSC2*, *RB1*, *PTCH1*, *STK11*, and *FH*, according to the new ClinGen guidance, with the expectation of escalating VUS to pathogenic or likely PVs (LPVs). The ratio of VUS reclassification is presented with descriptions of representative cases.

## Materials and methods

### Study design and target variants

**Fig. 1:**
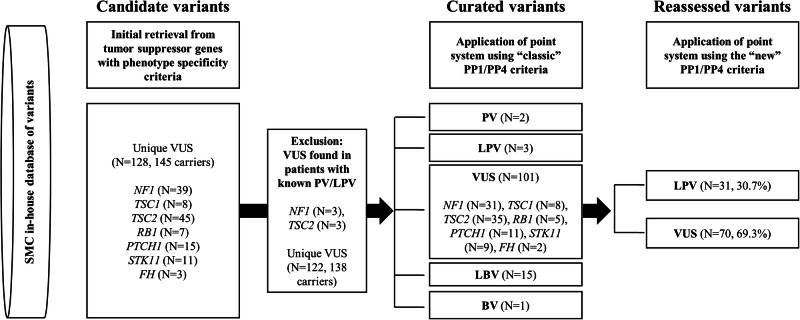
Variant reassessment workflow in this study. SMC, Samsung Medical Center; VUS, variant of uncertain significance; (L)PV, (likely) pathogenic variant; (L)BV, (likely) benign variant.

This study was approved by the Institutional Review Board of Samsung Medical Center (SMC), Seoul, Korea (approval numbers 2024-02-006 and 2025-06-084). The study was performed as a retrospective review of medical records and genetic variants previously detected from the routine clinical tests in a single tertiary general hospital. The processes of patient selection and variant filtering are illustrated in Fig. 1. The primary data source utilized was an in-house database of variants, which has collected all germline variants detected by Sanger sequencing and NGS conducted at the SMC since 2008. As all tests were conducted during routine clinical care, the determination of testing eligibility criteria was at the discretion of the clinicians. In SMC, all detected VUS were reported, irrespective of their potential for reclassification. The VUS in the SMC variant database underwent periodic reassessment for pathogenicity every three years as part of the variant reassessment program aimed at minimizing the number of VUS in the database. Independent of this routine reassessment program, in this study, the VUS in the database was cross-sectionally reassessed using the new ClinGen guidance, for the purpose of evaluating the effectiveness of the new guidance.

In December 2023, variants from seven target genes, *NF1*, *TSC1*, *TSC2*, *RB1*, *PTCH1*, *STK11*, and *FH*, which were remaining as VUS in the database, were initially selected. Patients with PV/LPVs detected as the cause of the disease were subsequently excluded. The clinical information of the patients included in the study, including phenotypes and family histories, was reviewed using electronic medical records.

### Variant assessment

The variants retrieved from the in-house database were annotated using ANNOVAR (version 2018, April 16) for databases of ClinVar [17] (version 2023.12.30), gnomAD [18] (Version 2.1.1), REVEL [19], and SpliceAI [20]. The pathogenicity of each variant was reassessed under manual review of annotated variants and phenotypes using the point-based system described in Tavtigian et al. [3] The application of the point-based system was performed two times for each variant, first, as a baseline, using classic PP1/PP4 criteria of ACMG/AMP guideline and second, using the new criteria provided in the ClinGen guidance [14]. Application of new PP1/PP4 criteria required the diagnostic yield values, which are transformed into points according to the predefined transition table. The diagnostic yield values of each gene were adopted from the mutational yield tables in GeneReviews entries (Table 1) [21**–**26], as suggested by the guidance. The phenotypic criteria used for PP4 application of both classic ACMG/AMP guidelines and point-based system are shown in Table 1. New PP1 criteria followed the Bayes point system outlined in the ClinGen guidance [14] and classic PP1 criteria were adopted from the ClinGen guidelines for other tumor suppressor genes, which require 3─4 meiosis for PP1 assignment [27, 28]. PM2 was applied when the Popmax filtering allele frequency (FAF) from gnomAD v.2.1.1 was 0. BS1 was applied for variants with FAF ≥ 0.03% and BA1 was applied for those with FAF ≥ 0.1%. PP3 was applied for variants with REVEL score ≥0.7 or Max SpliceAI score ≥0.2 and BP4 was applied for those with REVEL score <0.2 or Max SpliceAI score <0.1.

**Table 1:** Test methods used and Bayesian points derived from the diagnostic yield.

Gene	PP4 criteria	Test method	Diagnostic yield	PP4 Score	Reference
*NF1*	Two or more of the following features Six or more café-au-lait macules >5 mm in greatest diameter in prepubertal individuals and >15 mm in greatest diameter in postpubertal individuals Freckling in the axillary or inguinal regions Optic pathway glioma	RNA sequence analysis	85.4%	5	Evans et al. [37]
	Two or more neurofibromas of any type or one plexiform neurofibroma A distinctive osseous lesion, such as sphenoid dysplasia, anterolateral bowing of the tibia, or pseudarthrosis of a long bone	RNA sequence analysis + MLPA	95.8%	5	Evans et al. [37]
	Two or more Lisch nodules identified by slit lamp examination or two or more choroidal abnormalities (bright, patchy nodules imaged by optical coherence tomography/near-infrared reflectance imaging) A parent who meets the diagnostic criteria for NF1	DNA sequence analysis	80.7%	4.5	Bianchessi et al. [38]
*TSC1*	Two or more of the following major features Angiofibromas, cardiac rhabdomyoma, multiple cortical tubers, hypomelanotic macules, lymphangioleiomyomatosis, multiple retinal nodular hamartomas, renal angiomyolipoma, shagreen patch, subependymal giant cell astrocytoma, subependymal nodules, and ungual fibromas	DNA sequence analysis	25.4%	1.5	Northrup et al. [22]
		DNA sequence analysis + MLPA	26.0%	1.5	Northrup et al. [22]
*TSC2*	Same as the criteria for *TSC1*	DNA sequence analysis	66.8%	3.5	Northrup et al. [22]
		DNA sequence analysis + MLPA	69.0%	4	Northrup et al. [22]
*RB1*	Fundus examination required, suggestive criteria (leukocoria, strabismus, change in eye appearance, and reduced visual acuity)	DNA sequence analysis	82.0%^*^	5	Lohman et al. [23]
		DNA sequence analysis + MLPA	98.0%^*^	5	Lohman et al. [23]
*STK11*	Two or more PJS-type hamartomatous polyps of the gastrointestinal tract Mucocutaneous pigmentation	DNA sequence analysis	82.5%^*^	5	McGarraty et al. [24]
*FH*	Cutaneous leiomyoma Renal cell carcinoma	DNA sequence analysis	90.0%	5	Kamihara et al. [25]
*PTCH1*	Multiple basal cell carcinoma (>20 without family history, >5 with family history of first-degree relative)	DNA sequence analysis	67.5%^*^	3.5	Evans et al. [26]

^*^Average of the lower and upper bound values was used because the diagnostic yield was given as a range.

*NF1* neurofibromatosis type 1, *PJS* Peutz-Jeghers syndrome, *MLPA* multiplex ligation-dependent probe amplification.

### Test methods

Variants in seven target genes were detected from various types of panel tests and single gene sequencing tests, such as common cancer predisposition panel, pheochromocytoma, colorectal cancer, and epilepsy (Supplementary Table 1). For Sanger sequencing, all the coding exons were amplified by PCR using primers designed in-house. PCR products were sequenced on an ABI 3730xl DNA Analyzer (Applied Biosystems, Foster City, CA, USA) using a BigDye Terminator Cycle Sequencing Kit (Applied Biosystems). Sequences were analyzed using Sequencher (Gene Codes Corp., Ann Arbor, MI, USA) and compared to reference sequences. Sanger RNA sequencing was performed only for *NF1* based on the methods described in a previous publication [29].

NGS was performed with hybrid capture-based enrichment using a NovaSeq 6000 system (Illumina, San Diego, California, USA) or a NextSeq 550 system (Illumina). The reportable range for both Sanger sequencing and NGS was within 25 bp of each end of the exon.

Multiplex ligation-dependent probe amplification (MLPA) was performed using the SALSA MLPA P124-C3 TSC1 Kit (MRC Holland, Amsterdam, The Netherlands), the SALSA MLPA P046-D1 TSC2 kit (MRC Holland), and the SALSA MLPA P081-D1-P082-C2 NF1 kit (MRC Holland), according to the manufacturer’s instructions. The ABI 3730xl DNA Analyzer (Applied Biosystems) was used for capillary electrophoresis and GeneMarker software (SoftGenetics, State College, PA, USA) was used for the analysis of electropherogram.

## Results

### Summary of variant reassessment

**Fig. 2:**
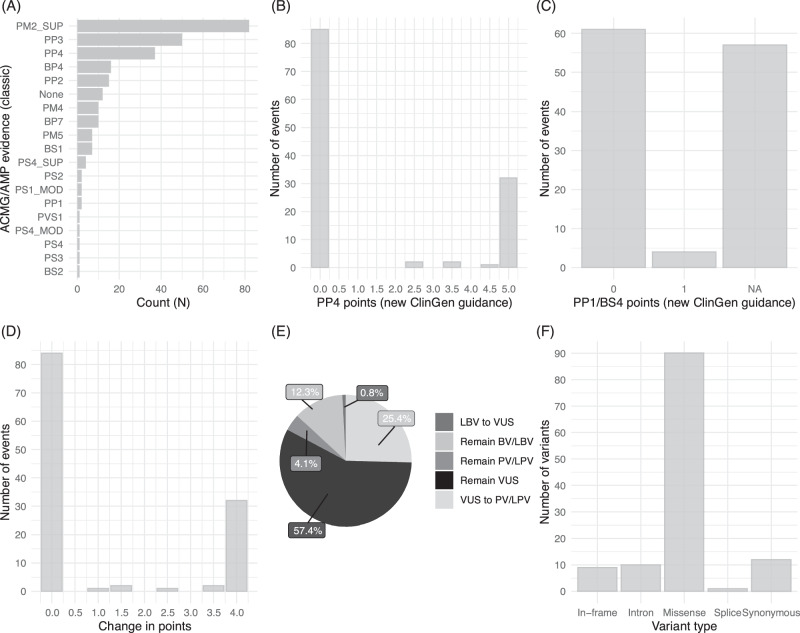
Overview of reclassification of 122 VUS. **A** Distribution of classic ACMG/AMP evidences assigned **B** PP4 points, **C** PP1/BS4 points assigned by new ClinGen guidance **D** Distribution of changes in points caused by the application of new ClinGen guidance **E** Overview of reclassification results **F** Types of variants reassessed. VUS, variant of uncertain significance; ACMG/AMP, American College of Medical Genetics and Genomics and the Association for Molecular Pathology; SUP, Supporting; MOD, Moderate; (L)BV, (likely) benign variant; (L)PV, (likely) pathogenic variant.

In total, 128 unique VUS from 145 carriers were retrieved from a database of variants. After excluding six VUS from patients with other causative PV/LPVs, 122 unique VUS from 138 carriers were curated (Fig. 1, Supplementary Table 1). Although all included variants were stored as VUS in the variant database, the application of a point system using an up-to-date database and classic PP1/PP4 criteria resulted in the reclassification of 21 variants into 2 PVs, 3 LPVs, 15 likely benign variants (LBVs), and 1 BV, resulting in 101 remaining VUS. The results of the subsequent application of the point system using the new PP1/PP4 criteria are listed in Table 2. Among the 101 remaining VUS, 31 (30.7%) were reclassified as LPV and 70 (69.3%) remained as VUS. One *TSC2* variant, which was classified as LBV according to the classic PP1/PP4 criteria, was returned to VUS based on the new PP1/PP4 criteria (TSC2_31). The classification of other variants as BV/LBV or PV/LPV by the classic PP1/PP4 criteria did not change with the application of the new PP1/PP4 criteria. Reclassification rates were diverse among the seven genes, with *STK11* showing the highest rate (8/9, 88.9%), whereas no VUS in *TSC1* was reclassified. The overview of evidence categories assigned by the classic ACMG/AMP guidelines and new ClinGen guidance, the result of reclassification, and variant types are shown in Fig. 2.

**Table 2:** Reclassification of VUS in seven tumor suppressor genes.

Classic PP1/PP4	New PP1/PP4	Total	*TSC1*	*TSC2*	*NF1*	*RB1*	*FH*	*PTCH1*	*STK11*
VUS	VUS	70	8	32	15	4	1	9	1
VUS	PV/LPV	31	0	3	16	1	1	2	8
PV/LPV	PV/LPV	5	0	1	3	1	0	0	0
LBV	VUS	1	0	1	0	0	0	0	0
BV/LBV	BV/LBV	15	0	5	2	1	1	4	2
Total		122	8	42	36	7	3	15	11
Reclassification rate (%) (VUS only)		30.7%	0.0%	8.6%	51.6%	20.0%	50.0%	18.2%	88.9%

*VUS* variant of uncertain significance, *(L)PV* (likely) pathogenic variant, *(L)BV* (likely) benign variant.

### Representative case of reclassified VUS in *FH* gene (FH_05)

A 58-year-old male was referred to a genetic counseling clinic with leiomyomas on his face and back. He had a history of left nephrectomy for renal cell carcinoma (RCC). His younger half brother also had a facial leiomyoma and was being treated for RCC in the SMC. Based on the clinical features and family history, the patient was highly suspected to have hereditary leiomyomatosis and renal cell cancer (HLRCC), but without genetic confirmation. The patient visited a dermatology clinic at another institution for a second opinion, and whole-exome sequencing revealed NM_000143.4:c.739 G > A, p.Glu247Lys in *FH*. The results have been published describing the variant as LPV without a clear description of evidence [30]. The variant was absent in gnomAD and had a REVEL score of 0.932. This variant has not been reported elsewhere, assigning PM2_Supporting, PP3, or PP4, which are insufficient for LPV classification.

**Fig. 3:**
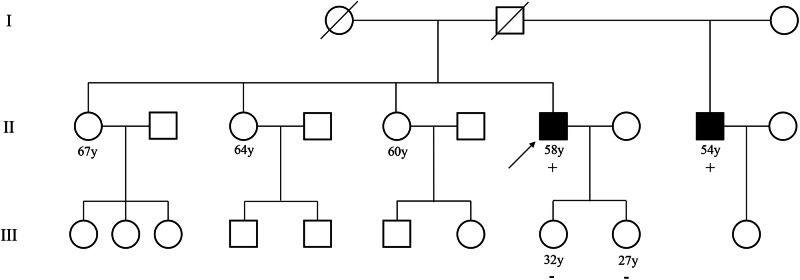
Pedigree of representative case of VUS detected in *FH* gene (FH_05). Arrow indicates the proband. Individuals who have done genetic test, their result is descripted below a square or circle, either “+” (variant detected) or “−” (variant not detected). The black square indicates that an individual has the HLRCC phenotype. VUS, variant of uncertain significance; HLRCC, hereditary leiomyomatosis and renal cell cancer.

During the genetic counseling conducted afterwards in the SMC, Sanger RNA sequencing was performed because of the terminal location of the variant in the exon; however, no evidence of aberrant splicing was observed. In addition, genetic tests for the patient’s younger half-brother and two asymptomatic daughters, aged 32 and 27 years-old, respectively, were performed, and segregation was identified enabling PP1 assignment (Fig. 3). However, according to the point system using the classic PP1/PP4 criteria, the point was still 4 (1 point each from PM2_Supporting, PP1, PP3, and PP4), which was insufficient for LPV classification. On the other hand, based on the new PP1/PP4 criteria, the PP4 score for FH was 5, based on a 90% diagnostic yield (Table 1), making the overall score of this variant 7 (5 points from PP4, 1 point each from PM2_Supporting and PP3), escalating the classification to LPV. Cutaneous leiomyoma is rare and highly suggestive of HLRCC [25], especially with concomitant RCC. As no other conditions mimic this phenotype, HLRCC caused by the FH variant could be considered a typical example of locus homogeneity. Instead, a PP1 score could not be assigned using the new PP1/PP4 criteria because of locus homogeneity.

### Representative case of reclassified VUS in *NF1* gene (NF1_20)

An infant visited the SMC for a work-up for neurofibromatosis. The patient had multiple (>30) café-au-lait spots sized >5 mm on arms, thighs, abdomen, and neck, along with freckles in both axilla. The patient’s clinical features suggested neurofibromatosis. Through genetic analysis, an in-frame deletion variant of *NF1*, NM_001042492.3:c.4253_4261del, p.Ile1418_Pro1421delinsThr, was detected by Sanger RNA sequencing, and no other VUS or PV/LPVs were noted. This variant was not observed in the population database (gnomAD), and there was no entry for this variant in ClinVar. Using the classic PP1/PP4 criteria, this variant had four points (PM2_Supporting, PM4, and PP4). However, when the new PP1/PP4 criteria were applied, five points were garnered for PP4 based on an 85.4% diagnostic yield (Table 1), resulting in a total of eight points enabling reclassification to LPV.

### Representative case of reclassified VUS in *PTCH1* gene (PTCH1_15)

**Fig. 4:**
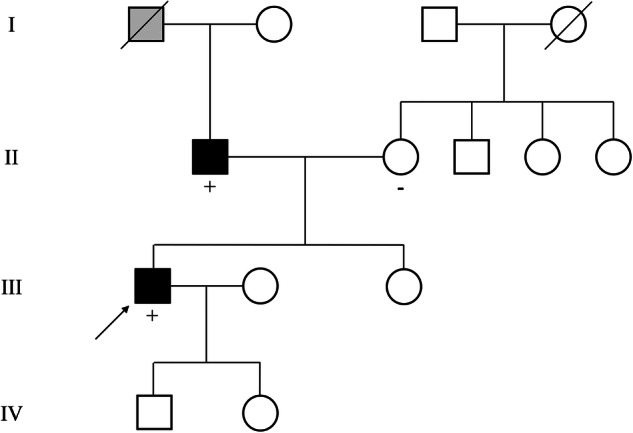
Pedigree of representative case of VUS detected in *PTCH1* gene (PTCH1_15). Arrow indicates the proband. Individuals who have done genetic test, their result is descripted below a square or circle, either “+” (variant detected) or “−” (variant not detected). A square or circle colored black indicates that an individual has a phenotype of BCNS, whereas the gray-colored square or circle indicates an individual’s phenotype is uncertain (alleged melanoma, in this case). BCNS, basal cell nevus syndrome; VUS, variant of uncertain significance.

A 38-year-old man presented with multiple and recurrent basal cell carcinomas (BCCs) on his palms, face, posterior neck, and left inner canthus that began in early childhood. The patient’s father had a history of recurrent BCCs primarily on his palms. NGS-targeting of genes related to germline cancer revealed a single VUS from *PTCH1*, NM_000264.5:c.3404 T > C, p.Leu1135Pro, without other VUS or PV/LPVs. The patient’s father harbored the same variant, which was not present in his mother (Fig. 4). This variant has not been reported in the literature, and its classification in ClinVar is conflicting (one LPV and one VUS). Using the classic PP1/PP4 criteria, the variant was assigned three points based on the absence of gnomAD (PM2_Supporting), a high REVEL score (0.913, PP3), and phenotype specificity (PP4). Based on the new PP1/PP4 criteria, the variant gained an additional 1 point from PP1 from the observed segregation, and 3.5 points, instead of 1, were garnered for phenotype specificity, reclassifying the variant as LPV (6.5 points).

### Representative case of reclassified VUS in *RB1* gene (RB1_08)

A pediatric patient with left-eye leukocoria underwent *RB1* Sanger sequencing. The patient’s ophthalmological evaluation and orbital computed tomography and magnetic resonance imaging findings suggested left unilateral retinoblastoma. A synonymous variant, NM_000321.3:c.861 G > A, p.Glu287= was detected in *RB1*, and no other VUS or PVs/LPVs were noted. This variant was absent in gnomAD and is located at the last nucleotide of exon 8, where in silico analysis suggested an aberrant splicing (SpliceAI score 0.74). Although exon 8 is located outside of functional domains, this variant had been reported in patients with retinoblastoma [31**–**33]. However, functional studies have not been performed. Genotype-phenotype correlation studies of retinoblastoma suggested missense or splice variants of *RB1* are related to incomplete penetrance [34, 35], conforming to the unilateral manifestation of the disease in this patient. MLPA revealed no exon deletion/duplication. Tissue sequencing could not be performed because of the loss to follow-up that happened shortly after the diagnosis of the proband. The application of the classic PP1/PP4 criteria classified this variant as VUS (4 points). However, using the new PP1/PP4 criteria, 5 points were garnered because of phenotype specificity, reclassifying this variant as LPV.

### Representative case of reclassified VUS in *STK11* gene (STK11_03)

A patient was tested using a colorectal cancer gene panel to confirm the clinical diagnosis of Peutz-Jeghers syndrome (PJS) and for subsequent genetic counseling. The patient was diagnosed with PJS during childhood based on a pigmented skin lesion on the lower lip and intussusception that required exploratory laparotomy. The patient also underwent right salpingo-oophorectomy for the enlargement of an ovarian polyp in adolescence. The patient underwent another laparotomy because of a second intussusception. The patient had multiple variable-sized polyps in the jejunum and proximal ileum, which were resected using intraoperative enteroscopy. The patient was also diagnosed with gastric-type adenocarcinoma of the cervix, requiring radical abdominal hysterectomy, and received concurrent chemoradiotherapy.

NGS revealed one VUS from *STK11* (NM_000455.5:c.500 T > C, p.Leu167Pro). This variant was absent from gnomAD, and no entry for this variant was observed in ClinVar. One publication listed the same variant found in a 17-year-old Korean boy with PJS, no description of the case or variant was provided [36]. The REVEL score for this variant was 0.9589. Using the classic PP1/PP4 criteria, the score for this variant was 3 (PM2_Supporting, PP3, and PP4). However, when the new PP1/PP4 criteria were applied, five points were obtained for PP4 based on an 82.5% diagnostic yield (Table 1), resulting in a total of 7 points enabling the LPV classification.

### Representative case of reclassified VUS in *TSC2* gene (TSC2_41)

A patient presented with shagreen patches on body, and brain MRI revealed cortical tubers and multiple subependymal nodules. VUS NM_000548.5:c.5068 G > C, p.Asp1690His was detected in *TSC2*. This missense variant, occurring at the last base of exon 39, was not found in population databases, whereas in silico analysis predicted that this variant would cause donor loss, and no functional studies, including RNA studies, have been conducted. Concurrent *TSC1* testing did not detect any PV/LPV or VUS, making the tuberous sclerosis testing scenario one of the locus homogeneity scenarios. As *TSC1* was excluded approximately 95% of the time, a causative variant was identified in *TSC2*. Therefore, phenotype specificity (PP4) points can be allocated to the *TSC2* variant ( + 7.0 points, which is capped at +5.0 points), reclassifying the VUS as LPV.

## Discussion

The ClinGen SVI has provided important refinements to the ACMG/AMP classification system to enhance consistency and transparency in the classification rationale. A new guidance from ClinGen SVI focused on two key evidence criteria: the co-segregation criteria (PP1/BS4) and the phenotype specificity criteria (PP4) [14]. The modifications made to these criteria were based on two insightful observations. First, PP4 evidence can be applied at point values substantially higher than previously thought as a supporting strength, particularly for disorders with highly specific phenotypes and high diagnostic yields. Second, the two evidence criteria, segregation and phenotype specificity, were not independent of each other.

In this study, we focused on syndromic disorders caused by seven tumor suppressor genes: *FH, NF1, PTCH1, RB1, STK11*, and *TSC1/2*. The patients in this study exhibited characteristic phenotypes that strongly suggest a genetic etiology. However, the identified variants in the genes of interest initially failed to reach LPV classification because of limited supporting evidence. Implementation of the new PP1/PP4 criteria facilitated the reclassification of these cases as LPVs. This reclassification offers patients opportunities for appropriate genetic counseling, family planning based on PGD, and potential targeted therapies. Considering that LPV criteria are defined by 90% posterior probability [3], LPV classification of the variants found in highly suspicious cases, such as those introduced in the Results section, would not be regarded as an overdiagnosis.

By choosing disorders with highly characteristic phenotypes, we focused more on PP4 than PP1/BS4 in this study. For these disorders, the prior probability of the gene’s role in the affected individual’s phenotype was high, leading to high diagnostic yield values (Table 1) and high scores for PP4 evidence.

Of the 101 VUS curated in this study, 31 (30.7%) were successfully reclassified as LPVs, all of which exhibited highly specific phenotypes and had notable PP4 values. In contrast, PP1/BS4 evidence was applicable to genes without locus homogeneity, specifically *TSC1/TSC2* and *PTCH1*, with less impact on the final classification than PP4 evidence. To the best of our knowledge, this is the first study to report the application of the new PP1/PP4 criteria across a large patient cohort.

As a limitation of this study, the gene selection was based on test results of a single institute and might not be comprehensive enough to be referenced by all institutions. For example, *SUFU* was not included in the analysis because of the lack of diagnostic cases. However, institutions that commonly encounter *SUFU* variants could establish PP4 point of *SUFU* based on the estimated diagnostic rate, in the same way the points were derived for other genes. In addition, future investigations should explore different aspects of the new guidance, such as disorders with recessive inheritance or a set of cases with extensive pedigree information, to further assess their clinical utility.

Although we adopted the point-based system for the application of the new guidance, it is described in the guidance that if the laboratory is using the previously described six-level evidence scheme of ACMG/AMP guideline (pathogenic very strong, strong, moderate, and supporting and benign strong and supporting), then the point values can be converted to those descriptive levels using the conversion table provided in the guidance [14]. For example, PP4_Strong and PP1_Supporting could be allocated instead of 5.0 points from the combined PP4 and PP1 evidence. This flexibility allows for the broader adoption of the new PP1/PP4 criteria within existing practices, ultimately benefiting more patients through an accurate diagnosis.

In conclusion, the application of the new PP1/PP4 criteria enabled the reclassification of a significant number of variants previously classified as VUS, despite the clinical circumstances indicating LPV classification. These new recommendations are expected to substantially enhance the accuracy of variant classification, thereby reducing the number of otherwise inevitable VUS classifications.

## Supplementary information

Supplementary Information

Supplemenatary Table 1

DNA Variant HGVS Nomenclature Verification

## Data Availability

The data that support the findings of this study are available upon request. Requests can be initiated by contacting the corresponding author by email (miaeyaho.jang@samsung.com). Data requests will be reviewed by the corresponding author and will be made available assuming the intent is to advance research, that there are no patient privacy or safety concerns, and that the data will not be made open access.
