# Supplementary material for: Reassessment of variants of uncertain significance in tumor suppressor genes using new ClinGen PP1/PP4 criteria guidance
Source: Eur J Hum Genet. 2025 Jul 23;33(10):1368–75. doi: 10.1038/s41431-025-01911-z (PMC12480980; doi:10.1038/s41431-025-01911-z)
Supplement: Supplementary file 1 — Supplementary Information [file 41431_2025_1911_MOESM1_ESM.docx]

**SUPPLEMENTARY INFORMATION**

**Supplementary Table 1**

**Text summary:** List of cases in this study, and their ACMG/AMP classification results with PP1/PP4 criteria guidance.

**File format:** .xlsx
